# Association between Augmented Renal Clearance and Inadequate Vancomycin Pharmacokinetic/Pharmacodynamic Targets in Chinese Adult Patients: A Prospective Observational Study

**DOI:** 10.3390/antibiotics11070837

**Published:** 2022-06-22

**Authors:** Jinjin Zhao, Yaxin Fan, Minjie Yang, Xiaoyu Liang, Jufang Wu, Yuancheng Chen, Beining Guo, Huifang Zhang, Ruilan Wang, Fengying Zhang, Jingqing Hang, Huayin Li, Jing Zhang

**Affiliations:** 1 Institute of Antibiotics, Huashan Hospital, Fudan University, Shanghai 200040, China; 20111220031@fudan.edu.cn (J.Z.); fanyaxin@fudan.edu.cn (Y.F.); 13111220011@fudan.edu.cn (M.Y.); victorliang0@gmail.com (X.L.); wujufang@huashan.org.cn (J.W.); chenyuancheng@huashan.org.cn (Y.C.); guobeining@huashan.org.cn (B.G.); 2 Key Laboratory of Clinical Pharmacology of Antibiotics, National Health Commission of People′s China, Shanghai 200040, China; 3 National Clinical Research Center for Aging and Medicine, Huashan Hospital, Fudan University, Shanghai 200040, China; 4 Phase I Clinical Research Center, Huashan Hospital, Fudan University, Shanghai 200040, China; 5 Emergency and Critical Care Department, Shanghai General Hospital, Shanghai Jiao Tong University School of Medicine, Shanghai 200080, China; zhanghuifang@sjtu.edu.cn (H.Z.); wangruilang@sjtu.edu.cn (R.W.); 6 Department of Pulmonary Medicine, Shanghai Putuo District People′s Hospital, Shanghai 200060, China; zhangfy74@sina.com (F.Z.); hangjq@hotmail.com (J.H.); 7 Department of Pulmonary Medicine, Zhongshan Hospital, Fudan University, Shanghai 200032, China; li.huayin@zs-hospital.sh.cn

**Keywords:** augmented renal clearance, vancomycin, pharmacokinetic/pharmacodynamic, area under the concentration–time curve to the minimal inhibitory concentration ratio, risk factor

## Abstract

This study aimed to examine the risk factors of augmented renal clearance (ARC) and the association between ARC and vancomycin pharmacokinetic/pharmacodynamic (PK/PD) indices in Chinese adult patients. A prospective, observational, multicenter study was conducted, and 414 adult patients undergoing vancomycin therapeutic drug monitoring (TDM) were enrolled. Clinical and PK/PD data were compared between ARC and non-ARC groups. Independent risk factors were examined using a multivariate logistic regression analysis. The ARC and augmented renal clearance in trauma intensive care (ARCTIC) scoring systems were evaluated. Eighty-eight of the enrolled patients (88/414, 21.3%) had ARC before vancomycin therapy. Patients with ARC were more likely to have subtherapeutic vancomycin PK/PD indices, including trough concentration (*p* = 0.003) and 24 h area under the concentration–time curve (AUC_24_) to minimal inhibitory concentration (MIC) ratio (*p* < 0.001). Male sex (OR = 2.588), age < 50 years (OR = 2.713), overweight (OR = 2.072), receiving mechanical ventilation (OR = 1.785), enteral nutrition (OR = 2.317), neutrophil percentage (OR = 0.975), and cardiovascular diseases (OR = 0.281) were significantly associated with ARC. In conclusion, ARC is associated with subtherapeutic vancomycin trough concentration and AUC_24_/MIC; therefore, higher than routine doses may be needed. Risk factors and ARC risk scoring systems are valuable for early identification.

## 1. Introduction

Vancomycin is a first-line antibacterial agent for the treatment of serious, life-threatening Gram-positive bacterial infections which has been researched for the optimization of therapy despite over 60 years of clinical use [1]. The pharmacokinetic/pharmacodynamic (PK/PD) index most relevant to vancomycin efficacy is the ratio of 24 h area under the concentration–time curve (AUC_24_) to the minimal inhibitory concentration (MIC) [2,3]. Trough serum concentration (C_min_) is a practical method of therapeutic drug monitoring (TDM) for intermittent infusion, whereas average steady-state concentration is often used for continuous infusion [4]. Both the 2009 vancomycin consensus guidelines from the IDSA and the 2020 updated guidelines [5,6] recommend that patients with unstable renal function receive vancomycin TDM. AUC_24_/MIC is recommended as a monitoring indicator instead of trough-only monitoring, and the recommended AUC_24_/MIC target was updated from 400 to 400–600, which has been found to be associated with improved clinical and bacteriological outcomes. Meanwhile, the 2020 updated guidelines from the Chinese Pharmacological Society [7] emphasize that patients with augmented renal clearance (ARC) should receive vancomycin TDM, and the therapeutic trough concentration target of 10–20 mg/L is still regarded as a recommended item.

ARC refers to enhanced renal elimination of circulating solute, which has been regarded as a frequent phenomenon in the critically ill [8] and defined as a creatinine clearance (CLcr) > 130 mL/min/1.73 m^2^ [9]. Previous studies have demonstrated that ARC results in subtherapeutic concentrations when standard dosage guidelines are followed, which might be associated with poor clinical outcomes [10,11,12,13,14]; thus, close observation and aggressive dosing strategies are needed. As vancomycin is primarily excreted via the kidney, hyperrenal function can significantly affect its elimination. It is of great importance to study the association between ARC and the vancomycin PK/PD indices. The effect of ARC on subtherapeutic vancomycin trough concentrations is well established [15,16,17]; however, evidence on how ARC affects vancomycin AUC_24_/MIC and treatment outcomes remains limited.

Previous evidence suggests that younger patients without comorbidities or organ dysfunction are more likely to exhibit ARC [9,18], those to whom less clinical attention might be paid; thus, early recognition of ARC remains clinically challenging. Several risk factors associated with ARC have been reported in which younger age has mostly been confirmed [9,19,20,21,22,23]. Other related factors include male sex [19,22,23], trauma [20,21,22,23], mechanical ventilation [23,24], high diastolic blood pressure [25], elevated cardiac index [22], and febrile neutropenia [26], which were not confirmed in all studies. For early recognition of ARC, Udy et al. [22] developed an ARC scoring system in ICU patients with sepsis or trauma based on the risk factors of age < 50 years (6 points), presence of trauma (3 points), and sequential organ failure assessment (SOFA) score ≤ 4 (1 point). Considering the impracticality of the SOFA score, Barletta et al. [27] developed the augmented renal clearance in trauma intensive care (ARCTIC) scoring system with a serum creatinine (SCr) concentration < 0.7 mg/dL (3 points), male sex (2 points), and age (age < 56 years, 4 points; age 56–75 years, 3 points) as risk factors. An ARC score ≥ 7 points or an ARCTIC score ≥ 6 points was set as the operational threshold to identify high ARC risk. Nonetheless, data on the evaluation of these two systems are scarce, particularly in Asian populations.

Therefore, we performed a prospective, multicenter, observational study to analyze the risk factors of ARC in adult patients and the impact of ARC on vancomycin PK/PD. In addition, we evaluated the ARC and ARCTIC scoring systems to determine whether they were suitable as screening tools.

## 2. Results

### 2.1. Patient Enrollment and Characteristics

A total of 414 adult Chinese patients with Gram-positive infections were enrolled in the study, including 88 ARC patients and 326 non-ARC patients. The median age of the population was 61 years (IQR, 49–74 years), and 277 patients (66.9%) were male. The proportions of cardiovascular disease (14.8% vs. 43.9%, *p* < 0.001), diabetes mellitus (8.0% vs. 17.8%, *p* = 0.024), stroke (14.8% vs. 25.8%, *p* = 0.031), and malignancy (35.2% vs. 24.2%, *p* = 0.038) in the ARC group were significantly lower than in the non-ARC group. The baseline median CLcr was 92 (IQR, 61–121) mL/min/1.73 m^2^ on the whole, with 159 (IQR, 144–193) and 78 (IQR, 55–101) mL/min/1.73 m^2^ in the ARC and non-ARC groups, respectively. Among the 414 patients, 252 (60.9%) were critically ill, with no significant difference (*p* = 0.549) between the two groups (Table 1).

### 2.2. ARC Patients and Risk Factors for ARC

Eighty-eight of the enrolled patients (88/414, 21.3%) were identified to have ARC before vancomycin therapy. Among critically ill patients, 56 (56/252, 22.2%) patients had ARC (Table 1) before vancomycin therapy.

After multivariate adjustment, sex (male or female), age (< 50 or ≥ 50 years), BMI (≤ 24 or > 24), presence of cardiovascular disease (yes or no), receiving mechanical ventilation (yes or no), receiving enteral nutrition (yes or no), and neutrophil percentage level were included in the final model. Male sex (OR, 2.588 [95% CI, 1.388–4.825]), age < 50 years (OR, 2.713 [95% CI, 1.548–4.754]), being overweight (OR, 2.072 [95% CI, 1.185–3.625]), receiving mechanical ventilation (OR, 1.785 [95% CI, 1.002–3.181]), and receiving enteral nutrition (OR, 2.317 [95% CI, 1.185–4.528]) were positively associated with ARC (*p* < 0.05). The presence of cardiovascular disease (OR, 0.281 [95% CI, 0.144–0.550]) and neutrophil percentage (OR, 0.975 [95% CI, 0.959–0.991]) were negatively associated with ARC (*p* < 0.05) (Table 2).

### 2.3. Treatment Outcomes and Microbiological Analysis

Of the 414 patients, 321 (77.5%) were successfully treated with vancomycin, and 93 (22.5%) had failed treatments with vancomycin. There was no significant difference in the efficacy (clinical, microbiological, and comprehensive) between the ARC and non-ARC groups (Table S1 in Supplementary Materials).

A total of 414 strains of Gram-positive clinical isolates were collected prior to vancomycin treatment. *Staphylococcus* spp. (321/414, 77.5%) were the most frequently encountered pathogens, followed by *Enterococcus* spp. (71/414, 17.1%) and *Streptococcus* spp. (18/414, 4.3%). Overall, 180 methicillin-resistant *Staphylococcus aureus* (MRSA) isolates accounted for 43.5% of the total isolates. No significant differences were found in the MIC distribution between the ARC and non-ARC groups, including vancomycin insensitivity (Table S2).

### 2.4. Associations between ARC and Vancomycin PK/PD Indices

**Figure 1:**
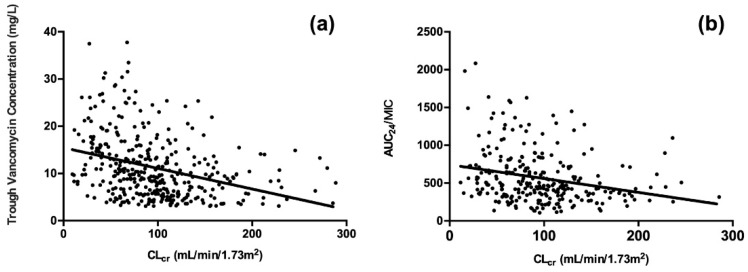
Correlations between vancomycin PK/PD indices and estimated creatinine clearance. (**a**): Correlation between steady-state trough vancomycin concentration and estimated creatinine clearance [r = −0.389, *p* < 0.001]; (**b**): Correlation between AUC_24_/MIC and estimated creatinine clearance [r = −0.287, *p* < 0.001]). Linear regression lines (solid) have been fitted to the data points; CLcr: estimated creatinine clearance (calculated by the Cockcroft–Gault formula); AUC_24_: area under the curve from 0 to 24 h; MIC: minimum inhibitory concentration.

Vancomycin steady-state trough concentration (r = −0.389, *p* < 0.001) and AUC_24_/MIC (r = −0.287, *p* < 0.001) were negatively correlated with CLcr (Figure 1).

As shown in Table 3, the initial daily dose was higher in the ARC group (*p* < 0.001), but the PK/PD indices (including steady-state trough concentration, AUC_24_, and AUC_24_/MIC) were significantly lower than those of the non-ARC group (*p* < 0.05). More descriptive statistics for initial daily dose of vancomycin were showed in Table S4. In order to remove the possible effect of vancomycin dose on renal function, we corrected the PK/PD values for initial daily dose. The results showed that the corrected PK/PD indices in the ARC group were still significantly lower than those of the non-ARC group (*p* < 0.05). The proportion of C_min_ values below the recommended targets (<10 mg/L) was significantly higher in the ARC group (71.6%) than in the non-ARC group (53.7%) (*p* = 0.003). The proportion of AUC_24_/MIC values below the recommended targets (<400) was also significantly higher in the ARC group (63.6%) than in the non-ARC group (33.1%) (*p* < 0.001). The results indicated that ARC was associated with subtherapeutic PK/PD indices and might be a risk factor for subtherapeutic exposure. There was no difference in the proportion of target achievement (C_min_ 10–20 mg/L, AUC_24_/MIC 400–600) between the two groups.

ROC analysis was performed to examine the accuracy of CLcr in predicting inadequate C_min_ (<10 mg/L) and AUC_24_/MIC (< 400). For C_min_, the area was 0.691 (95% CI, 0.638–0.743; *p* < 0.001), and the optimal cutoff, sensitivity, and specificity were 90.49 mL/min/1.73 m^2^, 66.4%, and 69.3%, respectively. For AUC_24_/MIC, the area was 0.626 (95% CI, 0.558–0.695, *p* < 0.001), and the optimal cutoff, sensitivity, and specificity were 85.30 mL/min/1.73 m^2^, 67.7%, and 53.6%, respectively (Figure S1). These results indicate that the single CLcr indicator performed poorly in predicting inadequate PK/PD index values, and a comprehensive predictive evaluation system should be considered for clinical use.

### 2.5. Evaluation of ARC Scoring Systems

The ARC score was calculated in the subset of critically ill patients (*n* = 252; 56 in the ARC group and 196 in the non-ARC group), while the ARCTIC score was calculated in the subset of trauma patients (*n* = 30; 9 in the ARC group and 21 in the non-ARC group). The proportion of patients with high-risk scores (ARC score ≥ 7, ARCTIC score ≥ 6) in the ARC group was significantly higher than that in the non-ARC group (both *p* < 0.001) (Table S3).

When CLcr ≥ 130 mL/min/1.73 m^2^ was used as the gold standard for diagnosing ARC, the evaluation ability of the ARC risk scoring system to identify ARC is shown in Table 4. An ARC score ≥ 7 had a sensitivity, specificity, PPV, NPV, and consistency rate of 0.589, 0.786, 0.440, 0.870, and 0.742, respectively. An ARCTIC score ≥ 6 had a sensitivity, specificity, PPV, NPV, and consistency rate of 0.889, 0.952, 0.889, 0.952, and 0.933, respectively (Table 4).

In critically ill patients, an ARC score of ≥7 performed well in predicting subtherapeutic C_min_ (OR, 5.431 [95% CI, 2.740–10.764]; *p* < 0.001) and AUC_24_/MIC (OR, 1.998 [95% CI, 1.061–3.766]; *p* = 0.009) (Table 5). In trauma patients, an ARCTIC score of ≥ 6 performed well in the prediction of subtherapeutic AUC_24_/MIC (*p* = 0.013). Regarding inadequate C_min_, although the proportions seemed to be different (88.9% vs. 57.1%) between the ARCTIC score ≥ 6 and ARCTIC score < 6 groups, statistical significance was not found (*p* = 0.178) (Table 6).

## 3. Discussion

To our knowledge, this is the first prospective, multicenter study examining the risk factors of ARC and evaluating the ARC risk scoring systems in an Asian population with a relatively large sample size (*n* = 414). More importantly, we demonstrated that ARC led to subtherapeutic vancomycin AUC_24_/MIC (<400), which served as the recommended PK/PD index of the latest guidelines [6]. Therefore, more attention should be paid to ARC patients, and individualized dose adjustments should be performed for this population.

Considering the effect of vancomycin dose on renal function, it was necessary to normalize the dose in PK/PD analysis. After the dose correction, we still found that ARC was associated with lower PK/PD indices (including C_min_, AUC_24_, and AUC_24_/MIC). Previous studies have focused on the association between ARC and vancomycin trough concentration [15,16,17], mainly because the previous consensus guidelines in 2009 [5] recommended the use of trough monitoring (target: 15–20 mg/L) as a surrogate marker of AUC_24_/MIC (target: >400) for ease of monitoring and simplifying dose adjustments. However, with more evidence about the increasing risk of nephrotoxicity from trough monitoring using these targets and the popularity of AUC calculation, the latest consensus guidelines in 2020 recommend AUC_24_/MIC (target: 400–600) as the monitoring indicator instead of trough-only monitoring [6]. To date, few studies have focused on the association between ARC and AUC_24_/MIC indices. Chen et al. [28] found that AUC_24_/MIC < 400 was more common in pediatric ARC patients from a retrospective cohort of 470 critically ill children. The present study provided evidence in adults with ARC, which is of great value for guiding vancomycin monitoring and individualized medication. Nonetheless, it should be noted that the AUC_24_/MIC target of 400–600 is mainly recommended for suspected or definitive serious MRSA infections, with an assumed vancomycin MIC of 1 mg/L [6], and there is no generally accepted PK/PD target for other bacteria. Considering that MRSA isolates accounted for 43.5% and that the vancomycin MIC_50_ was 1 mg/L in the present study, it is reasonable to use this target.

In this study, we found that 21.3% (88/414) of adult patients with infections were identified to have ARC, which was comparable to that reported in previous studies (14–80%) [29]. Previous studies focused more on subsets of critically ill patients, including mixed populations of both ICU and non-ICU patients, and found a similar proportion of ARC patients among the ICU patients (22.2%, 56/252), suggesting that ARC needs to be taken seriously in any hospital department [24,30].

Notably, evidence that ARC affects the treatment efficacy has not been confirmed in all studies. Huttner et al. [21] found that ARC strongly predicted undetectable plasma concentrations of β-lactam antibacterial agents but found no link between ARC and clinical failure in 100 critically ill patients. Udy et al. [19] also found no association between ARC and clinical outcomes in 254 critically ill patients with severe sepsis who received β-lactam therapy. In the present study, we failed to find associations between ARC and vancomycin treatment outcomes, either in terms of clinical outcomes or bacterial clearance. This phenomenon may be due to the fact that ARC patients tend to have relatively mild organ dysfunction and strong compensatory reserves [19,21]. Although evidence of the direct correlation between ARC and vancomycin treatment failure is limited, ARC has been shown to increase the likelihood of a negative outcome, owing to the clear association with subtherapeutic concentrations and lack of PK/PD target attainment [15,16,17,28].

The present study incorporated rich clinical indicators to identify risk factors for ARC. Finally, we found that patients with ARC tended to be male, younger, overweight, receiving mechanical ventilation, receiving enteral nutrition, with lower neutrophil percentage and with no cardiovascular disease. Among these factors, male sex [19,22,23], age < 50 years [9,19,20,21,22,23], and mechanical ventilation [23,24] were identified as significant risk factors for ARC. Notably, the present study is the first to report that overweight patients are more likely to manifest ARC. This may be because overweight can influence several physiological processes and, consequently, enhance renal function [31]. As being overweight and obese have become major public health issues worldwide, the risk of ARC and subtherapeutic vancomycin status should be taken seriously. In addition, a few studies have reported that patients receiving enteral nutrition are more likely to develop ARC. A reasonable explanation is that enteral nutrition leads to increased protein loading, which causes an increase in glomerular filtration in response. Nonetheless, these results require further confirmation.

Hirai et al. [26] first reported febrile neutropenia as a significant risk factor for ARC in 109 Japanese pediatric patients. In the present study, however, we only found that febrile neutropenia was associated with ARC in univariate analysis (*p* = 0.015), due to the small proportion of febrile neutropenia patients (14/414, 3.4%). The multivariate analysis results preliminarily suggested that a decreased neutrophil percentage might be a potential risk factor for ARC. In addition, this study found that the Charlson comorbidity index of the ARC group was higher than that of the non-ARC group, with borderline statistical significance (*p* = 0.050). We attempted to use this variable in the multivariate analysis and found no statistical significance; therefore, we chose to include specific diseases in the model. Finally, we found that cardiovascular disease was a related factor that has rarely been reported. Cardiovascular disease is a combination of several specific diseases with different mechanisms in which high diastolic blood pressure [25] or elevated cardiac index [22] can lead to ARC, resulting in increased renal blood flow. These results could only serve as preliminary warnings but are still of great significance for early clinical detection.

The role of individual risk factors in the early clinical detection of high-risk ARC patients is relatively limited. Reasonable ARC risk scoring systems are practical tools for screening high-risk patients with ARC. The present study evaluated two widely used ARC risk scoring systems and attempted to select suitable subgroups from the present population. We found that the ARCTIC system had high sensitivity and specificity and performed well in the prediction of subtherapeutic AUC_24_/MIC in patients with trauma. The ARC score performed well in the prediction of subtherapeutic C_min_ and AUC_24_/MIC but had poor sensitivity for the prediction of high-risk ARC in ICU patients. This was partly because the ARC scoring system was developed for patients with sepsis and trauma in intensive care, which presented a certain difference compared to the composition of ICU patients in the present study. In general, both systems have clinical application value because of the sensitive identification of subtherapeutic PK/PD targets. For the prediction model, it is important to keep the evaluated population consistent with the applicable model population. Possibly, a more general prediction system can be developed in the future for use in several departments, rather than being limited to the ICU.

This study has several limitations. First, the use of estimated CLcr carries more bias than measured urinary CLcr, particularly for elevated CLcr [32]. Importantly, the ARC scoring systems used measured urinary CLcr [22,27], which might introduce a substantial bias, as it has been shown that estimators of renal function are imprecise and biased with regard to measured CLcr, especially in the ARC range. Considering that 8- or 24-hour urine collection was not practical in the present study, we selected the Cockcroft–Gault formula, which might be the best method for CLcr estimation in the ARC population [32,33]. Secondly, some comorbidities were not included in the multivariate analysis model, owing to collinearity or limited statistical power, which may have missed some risk factors. Additionally, this study regarded age and BMI as dichotomous variables when screening independent risk factors for ARC. Although the results indicated that younger age and higher BMI may increase the risk of ARC, we might lose some information by using dichotomous variables rather than continuous variables in the multivariate logistic regression model. Nonetheless, the present study only served as a preliminary screening for comorbidities, and more conclusions need to be explored in follow-up studies. Thirdly, the small sample size of trauma patients may have affected the statistical power of the evaluation of the ARCTIC scoring system. In addition, the current “peak–trough” sampling strategy could be further optimized for the estimation of AUC_24_. Uster. et al. [34] reported that the optimal single-sample timepoints were identified between 2 and 6.5 h post-dose; sampling of trough concentrations might result in a higher imprecision. Adding a second sample between 4.5 and 6.0 h improved the predictive performance. The optimal two-sample strategy outperformed the “peak–trough” strategy, but the differences were minor. Therefore, the classical “peak–trough” sampling method used in this study might result in acceptable predictions. Model-informed sampling strategies should be more considered in further study design.

## 4. Materials and Methods

### 4.1. Study Design

We conducted a prospective, multicenter, observational study in 17 teaching hospitals across China from September 2012 to July 2020. The study protocol and informed consent form were reviewed and approved by the Ethics Committee of Huashan Hospital, Fudan University (No.2012-140/No.2017-255), and the study was performed in accordance with the principles of the Declaration of Helsinki and the Good Clinical Practice guidelines. This study was approved by other sub-centers. Informed consent was obtained from all patients before enrollment, and the data were anonymized. This study was registered in the Chinese Clinical Trial Registry at www.chictr.org.cn (accessed on 4 September 2017) (accession number: ChiCTR-OPC-16007920/ChiCTR-OPC-17012567).

### 4.2. Study Population and Data Collection

Patients were eligible for enrollment if they were adults with Gram-positive bacterial infections based on both clinical (symptoms, signs, and laboratory tests) and microbiological (e.g., blood, sputum, and urine culture) evidence. They had at least 5 days of vancomycin therapy and underwent vancomycin TDM. Patients who fulfilled the following criteria were excluded: (1) received any other antimicrobial therapy effective for Gram-positive bacteria for more than 24 h within 72 h before enrollment; (2) Gram-positive bacterial colonization; (3) pregnant or lactating women; (4) co-administration of nephrotoxic antibacterial agents; (4) patients with renal replacement therapy; and (5) had missing data on age, sex, weight, height, and baseline SCr.

Demographic characteristics, comorbidities (including the Charlson comorbidity index), primary infection sites, exposures, laboratory findings, and combination therapies were collected in a uniform case record form using electronic medical records. In this study, cardiovascular diseases included hypertension, coronary heart disease, rheumatic heart disease, congenital heart disease, myocardial infarction, and heart failure. Patients with a BMI ≥ 24 kg/m^2^ were defined as overweight, according to the Working Group on Obesity in China [35,36]. Patients admitted to the ICU were defined as critically ill, and the modified SOFA score was calculated.

### 4.3. ARC Evaluation

In this study, patients with baseline CLcr over 130 mL/min/1.73 m^2^ before vancomycin administration were identified to have ARC. CLcr was calculated using the Cockcroft–Gault formula [37]. The glomerular filtration rate (GFR) was estimated using the modification of diet in renal disease (MDRD) equation [38].

### 4.4. Vancomycin Administration and Sampling

The recommended initial regimen of vancomycin for adult patients with normal renal function is 15–20 mg/kg but no more than 2 g for a single-dose, intravenous infusion every 8–12 h. If the single dose exceeds 1 g, the recommended infusion time should be more than 1.5–2h. In critically ill patients, a loading dose of 25–30 mg/kg with an intravenous infusion time of at least 2 h should be considered. Adjustment of the individual dosing regimen was based on renal function and the results of TDM for treatment requirements. The recommended TDM target for vancomycin trough concentration was 10–15 mg/L for regular infections and 15–20 mg/L for critically ill patients with bloodstream infections, infective endocarditis, osteomyelitis, meningitis, pneumonia, severe skin and soft tissue infections, and so on.

For patients with normal renal function, serum samples were collected pre-dose (within 0.5 h) to determine the trough concentration and at 0.5–1 h post-dose to determine the peak concentration at the fourth or fifth dose. Serum samples were collected at the second dose in patients with GFR < 30 mL/min. Vancomycin TDM samples were assayed by fluorescence polarization immunoassay or chemiluminescence immunoassay, with a detection range of 3.00–100 mg/L.

### 4.5. Clinical Outcome Definition

Both clinical efficacy and microbiological eradication were considered in the assessment of vancomycin treatment outcome. Treatment success was defined as the eradication or presumed eradication of the baseline pathogens and no requirement of additional anti-microbial agents for Gram-positive bacteria within 7 days after the end of vancomycin treatment. Treatment failure was defined as no improvement in clinical symptoms, signs, and laboratory results after vancomycin treatment and/or persistent presence of baseline pathogens.

### 4.6. Microbiological Data and PK/PD Analysis

Clinical isolates of Gram-positive pathogens were collected prior to vancomycin treatment. The vancomycin MIC was verified using the agar dilution method, while the MIC data were interpreted according to the breakpoints in the Clinical Laboratory Standards Institute documents M07–A9 and M100–S24.

Individual AUC_24_ values were estimated using a Bayesian approach based on a previously developed vancomycin population PK model [39]. The model was a one-compartment population PK model; CLcr was the significant covariate of clearance (CL); and age was a significant covariate of volume of distribution (V).

According to the recommended targets from the latest guidelines [6], subtherapeutic vancomycin pharmacokinetic/pharmacodynamic targets were defined as C_min_ < 10 mg/L or AUC_24_/MIC < 400, while therapeutic vancomycin pharmacokinetic/pharmacodynamic targets were defined as C_min_ 10–20 mg/L and AUC_24_/MIC 400–600.

### 4.7. Statistical Analysis

All variables are summarized using descriptive statistics. The median and interquartile range (IQR) were calculated for continuous variables. Categorical data were summarized as counts and percentages. The Mann–Whitney U test was used for continuous variables, and the χ2 or Fisher′s exact test was used for categorical variables. A two-tailed value of *p* < 0.05 was considered statistically significant. SPSS statistics version 22.0 (SSPS Inc., Chicago, IL, USA) was used for the statistical analyses.

#### 4.7.1. Risk Factors for ARC

In the univariate analysis, the patients were divided into two groups based on the presence of ARC in order to determine the potential risk factors for ARC. All candidate variables with *p* values < 0.1 in the univariate analysis were included in the initial model, and a forward stepwise logistic regression analysis was performed to define significant risk factors for ARC. Only one correlated variable was selected for inclusion. The Hosmer–Lemeshow test was used to evaluate the model calibration. Results of multivariate analyses are presented as odds ratios (OR) and 95% confidence intervals (CI).

#### 4.7.2. Association between ARC and Vancomycin PK/PD Indices

Correlations between C_min_, AUC_24_/MIC, and CLcr were assessed using Spearman′s correlation coefficient. Patients were divided into ARC and non-ARC groups, and a univariate analysis was performed to determine the potential relationship between ARC and vancomycin PK/PD indices (below the targets/not below the targets, achieving the targets/not achieving the targets, above the targets/not above the targets). We corrected for dose using PK/PD values divided by initial daily dose (g). Receiver operating curve (ROC) analysis was performed to examine the accuracy of CLcr for predicting inadequate vancomycin PK/PD indices. The optimal cutoff value and corresponding sensitivity and specificity estimates were determined using the Youden index.

#### 4.7.3. Evaluation of ARC Scoring Systems

The ARC score was calculated in a subset of critically ill patients, whereas the ARCTIC score was calculated in a subset of trauma patients. The diagnostic performance of the ARC/ARCTIC scoring systems was evaluated using the model sensitivity, specificity, positive predictive value (PPV), negative predictive value (NPV), and consistency rate— which was defined as the percentage of correct predictions—using the scoring systems. Patients were divided into two groups based on the scores of the ARC risk scoring systems, and a univariate analysis was performed to determine the potential association between ARC score ≥ 7/ARCTIC score ≥ 6 and the vancomycin PK/PD indices (below the targets/not below the targets, achieving the targets/not achieving the targets, above the targets/not above the targets).

## 5. Conclusions

ARC was associated with subtherapeutic vancomycin C_min_ and AUC_24_/MIC and higher doses than routinely used, and TDM-guided dose optimization may be needed. Male sex, age < 50 years, being overweight, receiving mechanical ventilation, receiving enteral nutrition, and lower neutrophil percentage are potential risk factors for ARC. Patients with cardiovascular disease are less likely to develop ARC. ARC risk scoring systems are valuable for the early identification of high-risk ARC or subtherapeutic vancomycin status.

## Figures and Tables

**Table 1:** Demographics and analysis for ARC risk factors.

Characteristics	Total Patients (*n* = 414)	ARC (*n* = 88)	Non-ARC (*n* = 326)	*p* Value
**Demographics**				
Male sex	277 (66.9)	66 (75.0)	211 (64.7)	0.069
Age (years)	61 (49–74)	50 (33–60)	64 (53–76)	<0.001 *
Age < 50	109 (26.3)	40 (45.5)	69 (21.2)	<0.001 *
BSA (m^2^)	1.78 (1.67–1.91)	1.80 (1.66–1.93)	1.78 (1.67–1.90)	0.202
BMI (kg/m^2^)	22.0 (19.8–24.2)	23.2 (20.3–25.6)	21.6 (19.6–24.1)	0.025 *
Overweight ^a^	122 (29.5)	36 (40.9)	86 (26.4)	0.008 *
**Baseline renal function**				
SCr (μmol/L)	62 (46–85)	39 (31–46)	69 (55–94)	<0.001 *
CLcr (mL/min/1.73m^2^)	92 (61–121)	159 (144–193)	78 (55–101)	<0.001 *
eGFR (mL/min/1.73m^2^)	114 (80–153)	200 (170–244)	103 (70–128)	<0.001 *
**Comorbidities**				
Charlson comorbidity index	2 (1–3)	2 (0–2)	2 (1–3)	0.050
Cardiovascular disease	156 (37.7)	13 (14.8)	143 (43.9)	<0.001 *
Diabetes mellitus	65 (15.7)	7 (8.0)	58 (17.8)	0.024 *
Stroke	97 (23.4)	13 (14.8)	84 (25.8)	0.031 *
Trauma	30 (7.2)	9 (10.2)	21 (6.4)	0.224
Malignancy	110 (26.6)	31 (35.2)	79 (24.2)	0.038 *
**Exposures**				
Vascular catheter	295 (71.3)	66 (75.0)	229 (70.2)	0.382
Urinary catheter	280 (67.6)	63 (71.6)	217 (66.6)	0.371
Mechanical ventilation	129 (31.2)	34 (38.6)	95 (29.1)	0.088
Enteral nutrition	78 (18.8)	24 (27.3)	54 (16.6)	0.023 *
ICU admission	252 (60.9)	56 (63.6)	196 (60.1)	0.549
ICU duration	21 (12–36)	26 (16–2)	21 (10–34)	0.057
**Primary infection site**				
BSI	147 (35.5)	34 (38.6)	113 (34.7)	0.489
IE	8 (1.9)	1 (1.1)	7 (2.1)	1.000
Pneumonia	127 (30.7)	26 (29.5)	101 (31.0)	0.795
SSTI	29 (7.0)	4 (4.5)	25 (7.7)	0.479
UTI	22 (5.3)	0	22 (6.7)	0.006 *
CNS infection	18 (4.3)	7 (8.0)	11 (3.4)	0.076
IAI	31 (7.5)	10 (11.4)	21 (6.4)	0.120
**Laboratory indicators**				
Neutrophil percentage (%)	82.4 (73.9–88.0)	78.7 (72.0–86.9)	83.2 (74.5–88.6)	0.013 *
Febrile neutropenia	14 (3.4)	7 (8.0)	7 (2.1)	0.015 *
ALB (g/L)	37 (27–36)	32 (28–36)	32 (27–36)	0.736
ALT (U/L)	29 (18–54)	36 (22–85)	28 (16–49)	0.002 *
AST (U/L)	31 (20–53)	32 (22–64)	30 (19–51)	0.143
**Combination therapy**				
Loop diuretic	82 (19.8)	18 (20.5)	64 (19.6)	0.864
Dehydrating agent	28 (6.8)	10 (11.4)	18 (5.5)	0.053

Data are presented as the median (interquartile range) or *n* (%); *, *p* < 0.05; ^a^ overweight: defined as BMI ≥ 24 kg/m^2^; ARC: augmented renal clearance; BSA: body surface area; BMI: body mass index; SCr: serum creatinine; CLcr: estimated creatinine clearance (calculated by the Cockcroft–Gault formula); eGFR: estimated glomerular filtration rate (calculated by the modification of diet in renal disease equation); BSI: bloodstream infection; IE: infective endocarditis; SSTI: skin and soft tissue infection; UTI: urinary tract infection; CNS: central nervous system; IAI: intra-abdominal infection; ALB: serum albumin; ALT: alanine aminotransferase; AST: aspartate aminotransferase.

**Table 2:** Multivariate analysis of risk factors for ARC.

Characteristics	OR	95% CI for OR	*p* Value
Male sex	2.588	1.388–4.825	0.003 *
Age < 50 years	2.713	1.548–4.754	<0.001 *
Overweight ^a^	2.072	1.185–3.625	0.011 *
Cardiovascular disease	0.281	0.144–0.550	<0.001 *
Mechanical ventilation	1.785	1.002–3.181	0.049 *
Enteral nutrition	2.317	1.185–4.528	0.014 *
Neutrophil percentage	0.975	0.959–0.991	0.003 *

Hosmer–Lemeshow statistic for the final model, *p* = 0.326; *, *p* < 0.05; ^a^ overweight: defined as BMI ≥ 24 kg/m^2^; ARC: augmented renal clearance; BMI: body mass index.

**Table 3:** Vancomycin dosing and PK/PD analysis.

Characteristics	Total Patients (*n* = 414)	ARC (*n* = 88)	Non-ARC (*n* = 326)	OR	95%CI for OR	*p* Value
Initial daily dose (g/d)	2 (1–2)	2 (2–2)	2 (1–2)	3.238	1.952–5.369	<0.001 *
PK/PD values						
C_min_ (mg/L)	9.0 (5.0–14.1)	7.1 (3.9–10.6)	9.6 (5.3–15.3)	-	-	0.001 *
<10	238 (57.5)	63 (71.6)	175 (53.7)	2.174	1.303–3.628	0.003 *
10–20	132 (31.0)	21 (23.9)	111 (34.0)	0.607	0.353–1.043	0.071
>20	44 (10.6)	4 (4.5)	40 (12.3)	0.340	0.118–0.979	0.046 *
AUC_24_	409.9 (318.5–558.9)	357.7 (271.5–419.1)	423.8 (339.9–546.0)	-	-	<0.001 *
AUC_24_/MIC	457.4 (322.0–711.8)	360.5 (253.8–475.0)	494.7 (357.3–728.2)	-	-	<0.001 *
<400	164 (39.6)	56 (63.6)	108 (33.1)	3.156	1.813–5.495	<0.001*
400–600	110 (26.6)	17 (19.3)	93 (28.5)	0.585	0.282–1.214	0.083
>600	140 (33.8)	15 (17.0)	125 (38.3)	0.392	0.190–0.805	<0.001 *
PK/PD values corrected for dose ^a^						
C_min_ per dose	5.7 (3.6–10.2)	4.2 (2.5–5.9)	6.6 (4.2–11.5)	-	-	<0.001 *
AUC_24_ per dose	249.3 (185.3–358.1)	180.3 (161.5–215.0)	278.9 (208.6–397.1)	-	-	<0.001 *
AUC_24_/MIC per dose	287.5 (180.0–457.3)	180.2 (157.9–245.8)	309.7 (205.9–530.6)	-	-	<0.001 *

Data are presented as *n* (%) or median (interquartile range); *, *p* < 0.05; ^a^ PK/PD values corrected for dose using PK/PD values divided by initial daily dose (g); ARC: augmented renal clearance; C_min_: trough concentration; AUC_24_: area under the curve from 0 to 24 h; MIC: minimum inhibitory concentration.

**Table 4:** Evaluation of the abilities to identify ARC of the ARC risk scoring systems.

High-Risk Score	Scoring System	Gold Standard *		Sensitivity	Specificity	PPV	NPV	Consistency Rate
		ARC	Non-ARC					
ARC Score ≥ 7	Positive	33	42	58.9%	78.6%	44.0%	87.0%	74.2%
	Negative	23	154					
ARCTIC Score ≥ 6	Positive	8	1	88.9%	95.2%	88.9%	95.2%	93.3%
	Negative	1	20					

*, Gold standard: CLcr ≥ 130 mL/min/1.73 m^2^; ARC: augmented renal clearance; ARCTIC: augmented renal clearance in trauma intensive care; PPV: positive predictive value; NPV: negative predictive value.

**Table 5:** PK/PD analysis under the evaluation of the ARC scoring system.

	Critically Ill Patients (*n* = 252)	ARC Score ≥ 7 (*n* = 75)	ARC Score < 7 (*n* = 177)	OR	95%CI for OR	*p* Value
C_min_ (mg/L)	9.0 (5.0–14.1)	6.7 (3.8–9.8)	9.5 (5.3–15.0)	-	-	<0.001 *
<10	150 (59.5)	63 (84.0)	87 (49.2)	5.431	2.740–10.764	<0.001 *
10–20	73 (29.0)	10 (13.3)	63 (35.6)	0.278	0.134–0.580	0.001 *
>20	29 (11.5)	2 (2.7)	27 (15.3)	0.152	0.035–0.658	0.012 *
AUC_24_/MIC	476.7 (320.1–710.4)	380.1 (265.3–619.0)	486.2 (353.3–757.6)	-	-	0.093
<400	100 (39.7)	39 (52.0)	61 (34.5)	1.998	1.061–3.766	0.009 *
400–600	67 (26.6)	15 (20.0)	52 (29.4)	0.693	0.317–1.516	0.123
>600	85 (33.7)	21 (28.0)	64 (36.2)	0.717	0.351–1.464	0.210

Data are presented as *n* (%) or median (interquartile range); *, *p* < 0.05; ARC: augmented renal clearance; C_min_: trough concentration; AUC_24_: area under the curve from 0 to 24 h; MIC: minimum inhibitory concentration.

**Table 6:** PK/PD analysis under the evaluation of the ARCTIC scoring system.

	Trauma Patients (*n* = 30)	ARCTIC Score ≥ 6 (*n* = 9)	ARCTIC Score < 6 (*n* = 21)	*p* Value
C_min_ (mg/L)	8.5 (3.8–13.3)	5.6 (3.8–7.6)	9.2 (4.0–14.6)	0.178
<10	20 (66.7)	8 (88.9)	12 (57.1)	0.204
10–20	9 (30.0)	1 (11.1)	7 (33.3)	0.374
>20	2 (6.7)	0	2 (9.5)	1.000
AUC_24_/MIC	492.0 (303.1–781.2)	277.5 (208.2–397.8)	538.5 (379.8–910.2)	0.011 *
< 400	12 (40.0)	7 (77.8)	5 (23.8)	0.013 *
400–600	8 (26.7)	1 (11.1)	6 (28.6)	0.393
>600	10 (33.3)	1 (11.1)	10 (47.6)	0.100

Data are presented as *n* (%) or median (interquartile range); *, *p* < 0.05; ARC: augmented renal clearance; ARCTIC: augmented renal clearance in trauma intensive care; C_min_: trough concentration; AUC_24_: area under the curve from 0 to 24 h; MIC: minimum inhibitory concentration.

## Data Availability

The data presented in this study are available on request from the corresponding author.

## Supplementary Materials

The following supporting information can be downloaded at: https://www.mdpi.com/article/10.3390/antibiotics11070837/s1, Table S1: Treatment outcome analysis; Table S2: Microbiological analysis and vancomycin susceptibility; Table S3: High-risk score in ARC risk scoring systems: analysis between ARC and non-ARC groups; Table S4: Descriptive statistics for initial daily dose of vancomycin; Figure S1: ROC curve of the ability of CLcr to predict vancomycin PK/PD indices not up to standard.
