# Supplementary material for: Association between Augmented Renal Clearance and Inadequate Vancomycin Pharmacokinetic/Pharmacodynamic Targets in Chinese Adult Patients: A Prospective Observational Study
Source: Antibiotics (Basel). 2022 Jun 22;11(7):837. doi: 10.3390/antibiotics11070837 (PMC9312211; doi:10.3390/antibiotics11070837)
Supplement: Supplementary file 1 [file antibiotics-11-00837-s001.zip › antibiotics-1737487-supplementary.pdf]

Table S1. Treatment outcome analysis

**Characteristics**	**Total patients (N=414)**	**ARC (N=88)**	**Non-ARC (N=326)**	***P* value**
Clinical efficacy				
Success	321 (77.5)	70 (79.5)	251 (77.0)	0.611
Failure	89 (21.5)	17 (19.3)	72 (22.1)	0.575
Microbiological eradication				
Success	339 (81.9)	77 (87.5)	262 (80.4)	0.123
Failure	53 (12.8)	9 (10.2)	44 (13.5)	0.415
Comprehensive efficacy				
Treatment success	321 (77.5)	71 (80.7)	250 (76.7)	0.426
Treatment failure	93 (22.5)	17 (19.3)	76 (23.3)	0.426

Data are presented as n (%); ARC: augmented renal clearance.

Table S2. Microbiological analysis and vancomycin susceptibility

**Characteristics**	**Total patients (N=414)**	**ARC (N=88)**	**Non-ARC (N=326)**	***P* value**
**Pathogen**				
Staphylococcus spp.	321 (77.5)	71 (80.7)	250 (76.7)	0.426
S.aureus	230 (55.6)	45 (51.1)	185 (56.7)	0.347
MRSA	180 (43.5)	37 (42.0)	143 (43.9)	0.760
MSSA	50 (12.1)	8 (9.1)	42 (12.9)	0.333
CoNS	91 (22.0)	26 (29.5)	65 (19.9)	0.053
Enterococcus spp.	71 (17.1)	15 (17.0)	56 (17.2)	0.977
E.faecalis	35 (8.5)	8 (9.1)	27 (8.3)	0.809
E.faecium	34 (8.2)	7 (8.0)	27 (8.3)	0.921
Streptococcus spp.	18 (4.3)	1 (1.1)	17 (5.2)	0.139
**Vancomycin susceptibility**				
I/R	3 (0.7)	0	3 (0.9)	1.000
MIC>1	51 (12.3)	11 (12.5)	40 (12.3)	0.954
MIC_{50}	1	1	1	NA
MIC_{90}	2	2	2	NA

Data are presented as n (%); ARC: augmented renal clearance; MRSA: methicillin-resistant *Staphylococcus aureus*; MSSA: methicillin-sensitive *Staphylococcus aureus*. CoNS: coagulase negative staphylococci; I: intermediate; R: resistant; MIC: minimum inhibitory concentration; NA: not applicable.

Table S3. High risk score in ARC risk scoring systems analysis between ARC and Non-ARC groups

	**Critically ill patients (N=252)**	**ARC (N=56)**	**Non-ARC (N=196)**	***P* value**
ARC score ≥ 7	75 (29.8)	33 (58.9)	42 (21.4)	<0.001*

	**Trauma patients (N=30)**	**ARC (N=9)**	**Non-ARC (N=21)**	***P* value**
ARCTIC score ≥ 6	9 (30.0)	8 (88.9)	1 (4.8)	<0.001*

Data are presented as n (%); *, $P<0.05$; ARC: augmented renal clearance; ARCTIC: augmented renal clearance in trauma intensive Care.

Table S4. Descriptive statistics for initial daily dose of vancomycin

Initial daily dose (g/d)	Mean	SD	Percentiles				
			Minimum	25th	50th	75th	Maximun
Total patients (N=414)	1.68	0.54	0.25	1.00	2.00	2.00	4.00
ARC group (N=88)	1.97	0.42	1.00	2.00	2.00	2.00	3.00
Non-ARC group (N=88)	1.61	0.55	0.25	1.00	2.00	2.00	4.00

ARC: augmented renal clearance.

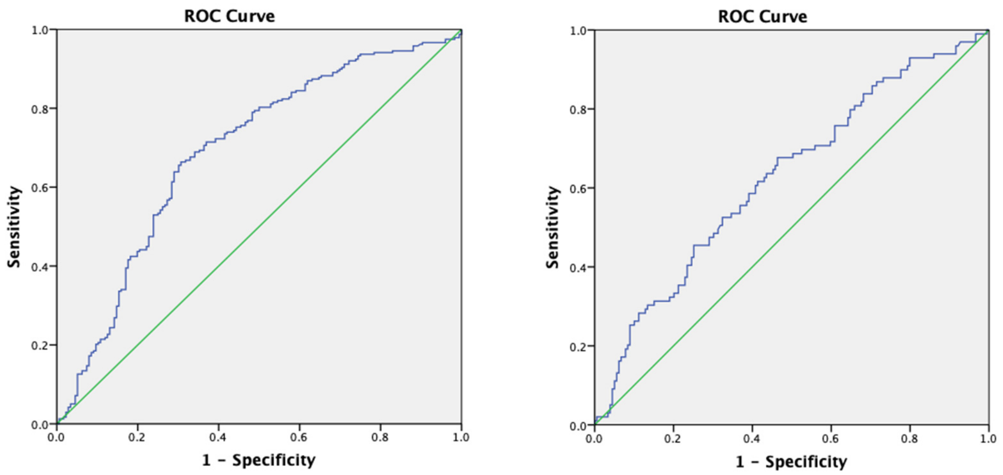

Figure S1. ROC curve of the ability of CLcr for predicting vancomycin PK/PD index not up to standard

Left: ROC curve of the ability of CLcr for predicting vancomycin C_{min}<10 mg/L (AUC: 0.691 [95% CI, 0.638–0.743], *P*<0.001; optimal cutoff value: 90.49mL/min/1.73 m^2, sensitivity: 0.664, specificity: 0.693);

Right: ROC curve of the ability of CLcr for predicting vancomycin C_{min}<10 mg/L (AUC: 0.626 [95% CI, 0.558–0.695], *P*<0.001; optimal cutoff value: 85.30mL/min/1.73 m^2, sensitivity: 0.677, specificity: 0.536)
